# A 3D Point Cloud Gesture Estimation Method Based on EdgeConv Reconstruction of Joint Features

**DOI:** 10.3390/s26144472

**Published:** 2026-07-14

**Authors:** Jiu Yong, Xiaomei Lei, Jianwu Dang, Zhenzhen Zhang

**Affiliations:** 1 The School of Electronic and Information Engineering, Lanzhou Jiaotong University, Lanzhou 730070, China; 2 School of Computer Science, Peking University, Beijing 100871, China; 3 College of Intelligence and Computing, Tianjin University, Tianjin 300072, China

**Keywords:** gesture estimation, 3D point cloud, edgeconv, feature reconstruction, inter-point relationship, feature guidance

## Abstract

**Highlights:**

- Using EdgeConv to enrich hand joint feature information effectively improves the performance of hand joint estimation.
- Reconstruct global features using EdgeConv in the initial joint estimation module, Construct local features centered around the modified joint through EdgeConv twice.

**Abstract:**

Gesture interaction allows users to interact with objects through natural hand movements without direct physical contact. However, in gesture interaction, the hand moves frequently with large variations in direction, and hand joints are prone to self-occlusion. The accuracy of existing 3D point cloud gesture estimation methods is insufficient to meet the requirements of natural gesture interaction. This article proposes a 3D point cloud gesture estimation method based on EdgeConv to reconstruct joint features. The method first converts depth maps into point cloud data to reduce the impact of viewpoint variations on depth map representation of the same gesture, thereby mitigating the effect on estimation accuracy. Then, the global features are reconstructed using EdgeConv in the initial joint estimation module so that the reconstructed features contain structural information between hand joints. Finally, local joint refinement is performed by constructing local features centered around the refined joint twice using EdgeConv. These features include reference information from within group points to group center points and structural information between joint points. Using EdgeConv to enrich hand joint feature information can effectively improve the performance of hand joint estimation. Comparative experimental analysis was conducted on the 3D point cloud datasets ICVL, NYU, and MSRA, and complex scene experimental analysis was conducted on the InterHand2.6M dataset. Furthermore, the development of virtual–real interaction applications was carried out. The experimental results show that the 3D point cloud gesture estimation method proposed in this paper has high accuracy, strong generalization and robustness, providing solid support for natural gesture interaction applications.

## 1. Introduction

Gesture interaction, as the core technology of embodied intelligence, allows users to interact with objects through hand movements without direct physical contact, and has been widely used in fields such as virtual assembly and autonomous driving [1]. According to different ways of obtaining gesture images, gesture interaction can be divided into two types: wearable-device-based and computer-vision-based. The gesture interaction technology based on wearable devices has high accuracy in tracking and estimating hand joints, but it is inconvenient to wear the device and may cause discomfort to the users participating in the interaction owing to wearing it for a long time. The gesture interaction technology based on computer vision can encode and extract gesture features from images captured by cameras containing hand regions, decode them to obtain gesture information, and design interaction semantics in specific interaction application scenarios based on the gesture information. Gesture estimation involves recognizing and processing images of the hand area to estimate joint information of the hand, making high-precision gesture estimation the cornerstone of natural gesture interaction. However, due to frequent hand movements, large changes in hand direction, easy self-occlusion, and the fact that the hand is hinged by numerous joints and has a variable posture, it is difficult to estimate hand joint information [2,3].

In recent years, a large number of gesture estimation algorithms based on deep learning have been proposed, which can be divided into two types according to input data: depth-map-based and color-map-based [4]. The gesture estimation algorithm based on color images is used to solve the highly nonlinear mapping from 2D color images to 3D spatial coordinates. This method requires a large amount of data sets and complex network structures to achieve a certain estimation accuracy, and has weak generalization ability. Depth image is an image that takes the distance (depth) from the image acquisition device to each point in the scene as a pixel value, containing spatial depth information. Convolution Neural Networks (CNNs) are widely used in gesture estimation algorithms based on depth maps [5,6,7]. Among them, the methods based on 2D CNNs are divided into two types: detection-based and regression-based. The regression-based method uses a 2D CNN to directly regress the 3D coordinates of hand joints from the depth map, while the detection-based method [8,9] uses a 2D CNN to extract a 2D heatmap representing the probability distribution of hand joints from the depth map. However, gesture estimation algorithms based on 2D CNNs cannot fully utilize the spatial depth information of depth maps, and some methods for converting depth maps into 3D data representations have been proposed [8]. Among them, the method of converting depth maps into voxel grid representations and applying 3D CNNs for gesture estimation has been extensively studied. However, the biggest limitation of this method is that as the voxel grid resolution increases, the parameters of the 3D CNN model will exponentially increase, resulting in low operational efficiency. Compared to sparse and discrete voxel representations, converting a depth map into a 3D point cloud of continuous spatial coordinate points can better represent the true gesture pose, indicating that this method preserves the detailed features of the opponent more completely during the data conversion process. However, due to the disorderliness and sparsity of point cloud data, it cannot be directly used as input for the estimation network. Usually, the collected 3D point cloud data needs to be converted into depth images from specific perspectives or into voxel data form in order to better define weight sharing convolution operations [1]. Taking HandFoldingNet as an example [9], EdgeConv and the FoldingNet decoder can be combined to form a 3D hand point cloud pose estimation network. By capturing local point cloud geometry through dynamic graph convolution and restoring hand mesh through folding modules, the basic estimation accuracy can be effectively improved. However, the existing EdgeConv only constructs a global point cloud dynamic map based on spatial distance, which cannot specifically explore the inherent anatomical topology and structural associations between finger joints and bone joints. On the one hand, EdgeConv does not have separate semantic parts, which can easily weaken the constraint features between joint points. On the other hand, HandFolding adopts a globally unified folding strategy, which lacks joint-oriented feature purification in the encoding stage. In sparse point clouds and hand self-occlusion scenes, joint features are easily overwhelmed by redundant skin points, making it difficult to accurately model inter-joint dependencies and ultimately leading to prediction biases such as finger misalignment [10]. Therefore, it is necessary to construct global and local graph convolutions for gesture joints and surrounding keypoints, directionally extract spatial and semantic structural information between joint points, strengthen prior constraints on skeletal topology, and achieve more stable and accurate 3D gesture estimation under occlusion and sparse point cloud conditions.

In summary, existing gesture estimation methods are unable to meet the accuracy requirements of natural gesture interaction applications due to frequent hand movements and large changes in direction, as well as the tendency of hand joints to self-occlusion. This article proposes a 3D point cloud gesture estimation method based on EdgeConv to reconstruct joint features. Based on the principle that using EdgeConv to reconstruct features in a Graph Convolution Neural Network (GCNN) takes into account the relationships between points, EdgeConv is used to reconstruct joint features. The reconstructed features take into account the information between hand joint points, which can effectively improve the estimation robustness of hand joint nodes. The specific contributions of this article are as follows:

1. During gesture interaction, the hand moves frequently with large changes in direction and movement amplitude, which can easily lead to self-occlusion and pose a huge challenge to hand joint estimation tasks. By applying EdgeConv theory to gesture estimation algorithms, hand joint features are reconstructed, and the reconstructed features consider the structural information between joints, effectively improving the accuracy of hand joint node estimation.
2. The depth map is converted into 3D point cloud data to reduce the impact of different depth maps for the same gesture due to different perspectives on the estimation results. Then, the gesture features are reconstructed by fusing the edge features of the point and all points in its neighborhood at the channel level as new features for that point. The reconstructed gesture features contain logical relationship information between points, effectively improving the performance of gesture estimation.
3. EdgeConv is used for feature reconstruction of global and local joint-level features, and the reconstructed features and folding decoding structures are used to construct global and local decoders for decoding the reconstructed features. The initial joint points with low accuracy and the corrected coordinate differences in the upper level estimated joint points relative to the true labels are obtained to estimate the hand joint node information. This approach not only considers global and local information, but also takes into account the structural information and position reference information between joint points, improving the robustness of hand joint point position estimation.

The remaining chapters of this article are as follows: Section 2 analyzes the research progress related to gesture estimation. Section 3 elaborates on the core framework structure and main modules of the method proposed in this article. Section 4 validates and analyzes the performance of our method from multiple dimensions. Section 5 delves into the innovation and limitations of the work presented in this article. Section 6 summarizes the entire text and explores future research directions.

## 2. Related Work

The robustness of gesture estimation algorithms determines the naturalness and experience of gesture interaction. The gesture estimation algorithms based on deep learning are divided into two categories: those based on depth maps and those based on color maps. The gesture estimation algorithm based on color maps is a highly nonlinear mapping from 2D color images to 3D spatial coordinates, with weak generalization ability. These algorithms require a large dataset and complex network structure to achieve certain accuracy in estimation results [11]. A depth map is an image that takes the distance (depth) from the image collector to each point in the scene as a pixel value, containing spatial depth information. With the release of low-cost and high-performance depth cameras such as Microsoft Kinect and Intel RealSense, a large number of gesture estimation algorithms based on depth maps have also been proposed.

Gesture estimation algorithms based on depth maps can be divided into two categories: fully supervised and weakly supervised. Due to the time-consuming and labor-intensive nature of labeling 3D gesture training data, a weakly supervised gesture estimation algorithm has been proposed [12]. However, the label data or gesture data generated based on semisupervised and unsupervised methods differ greatly from real data, and self-supervised methods heavily rely on the design of control functions, so weakly supervised gesture estimation algorithms have unstable estimation results. CNN is widely used in fully supervised methods, among which 2D-CNN-based methods are divided into regression-based methods and detection-based methods [13]. Based on regression, a 2D CNN is used to directly regress the 3D coordinates of hand joint points in depth maps. This method essentially establishes a highly nonlinear mapping from 2D input to 3D output, requiring a large amount of data and complex network structure to ensure the estimation accuracy of hand joint points. The detection-based method utilizes 2D CNN to extract the probability distribution of hand joint nodes in the 2D heatmap representation of the depth map, and calculates the probability that all pixels in the depth map are joint points, which increases the computational complexity of the algorithm. Due to the inability of 2D CNN algorithms based on depth maps to fully utilize spatial depth information, although some algorithms that convert depth maps into voxel grids and use 3D CNN for gesture estimation have been proposed, the voxel grid representation method based on depth maps using 3D CNN has inherent limitations. As the voxel grid resolution increases, the parameters of the 3D CNN model grow exponentially, consuming memory space [14]. In addition, spatial topology modeling and robust self-occlusion representation have always been the core difficulties that constrain the improvement of accuracy in 3D gesture estimation tasks. Traditional gesture perception methods mainly rely on techniques such as multi-view imaging, voxel convolution, and reflection transformation imaging [15]. These methods have significant shortcomings in spatial topology modeling and occlusion adaptability, making it difficult to adapt to unordered 3D point cloud input scenes [16]. For example, Pistellato et al. [17] proposed a lightweight reflection transformation imaging (RTI) acquisition scheme for mobile devices, which can complete data acquisition through only two ordinary smartphones. RTI uses scene markers to dynamically estimate the direction of moving light sources, and combines principal component analysis to compress massive reflection data. Combined with neural re-rendering models, it achieves surface reconstruction of objects in any lighting direction, breaking through the traditional RTI’s dependence on dedicated hardware and greatly improving the convenience and universality of imaging methods. However, this type of method is essentially based on the surface geometry and illumination fitting of 2D image sequences, which can only restore the surface morphology information of objects and cannot construct a complete 3D spatial topology structure. It lacks the ability to model the dynamic deformation and joint hierarchy relationship of non-rigid objects [18]. When facing complex scenes such as hand finger crossing, occlusion, and posture changes, this type of method is prone to feature loss and topological confusion, making it difficult to effectively meet the high-precision 3D gesture joint estimation requirements.

Compared to the sparse and discrete voxel representation, depth maps can be transformed into continuous point cloud representations of spatial coordinates. By using point cloud feature networks to extract hand point cloud data features, hand joint point localization can be achieved, which is more accurate than voxel representation methods [19]. Traditional neural network structures are unable to handle data with unordered members as the results also change when the input order of data members is altered. Changing the order of its member points in a point cloud does not change the point cloud data. Existing mainstream point cloud gesture estimation methods include PointNet [20], PointNet++ [21], Folding Net [22], Dynamic Graph Convolution Neural Networks (DGCNNs) [23], HandJoKe [24], etc. The gesture estimation algorithm based on point clouds needs to deal with the problem of unordered point members in point cloud data. For example, Ge et al. [25] used PointNet++ as a feature extractor to extract global features of gesture point clouds and directly regress the coordinates of hand nodes. Chen et al. [26] designed semantic segmentation networks and pose regression networks based on the PointNet++ structure, respectively. By integrating the joint-based semantic labels obtained from the semantic segmentation network into the input and output of the pose regression network, the accuracy of joint regression was increased. However, these methods only utilize global features, and the estimation accuracy cannot meet the requirements of natural gesture interaction. Li et al. [27] used Permutation Equivariant Layer (PEL) in residual networks to extract point level features as input for the point to pose voting framework. In order to better obtain 3D spatial information, Ge et al. [28] designed a stacked architecture with a symmetrical structure similar to an hourglass through skip connections in the feature extraction stage of the PointNet++ structure, achieving point-to-point feature extraction. Then, a multi-layer perception machine (MLP) was used to output point-by-point heatmaps and unit vectors reflecting the distance and direction from the input point to each joint, in order to calculate the residual coordinate difference at the joint level to estimate the position of the hand joint node. However, this method calculates the probability heatmap and direction vector of the entire point cloud relative to the joint, which has a huge computational load and consumes a lot of computing resources. Cheng et al. [9] used the PointNet++ global encoder and folding decoder to roughly estimate the initial joint points of the hand, and then constructed local features centered on the initial joint points to input into a self-designed local Folding decoder to obtain joint residual coordinates for estimating hand joints. However, this method did not consider the structural relationships between joint points, which further improved the accuracy of gesture estimation. HandJoKe [24] proposed a multimodal fusion framework that combines 2D depth maps with 3D point clouds. Based on a joint guided Transformer denoising mechanism, the framework filters out background redundant and noisy points in the point cloud, maintaining high accuracy even with low-quality point cloud input.

In summary, 3D point cloud data represents a series of 3D coordinate points distributed on the surface of the target. Point cloud data can better represent real gesture poses. However, due to the difficulty of using 3D point cloud data directly as input for estimation networks, researchers usually converted the collected point cloud data into depth images from specific perspectives or voxel data before PointNet was proposed, in order to better define weight sharing convolution operations. The use of EdgeConv to reconstruct features in GCNN takes into account the relationships between points, and the EdgeConv point cloud processing model can be effectively applied to gesture pose estimation tasks. This will be an important research direction for optimizing gesture pose estimation methods based on point cloud data.

## 3. Materials and Methods

**Figure 1:**
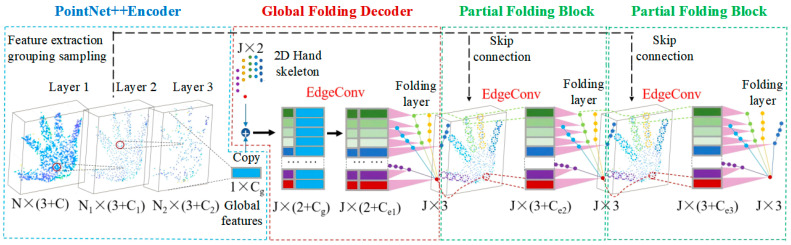
The method framework of this article.

Gesture estimation algorithms based on 2D CNNs cannot fully utilize the depth information in the depth map space, while gesture estimation algorithms based on voxel grids will occupy memory space and have low running efficiency as the grid resolution increases and the network model parameters increase. Gesture estimation algorithms based on point clouds tend to overlook the structural information between hand joint points, and the accuracy of gesture estimation needs to be improved. This paper proposes a 3D point cloud gesture estimation method based on EdgeConv to reconstruct joint features. EdgeConv is introduced to reconstruct the features of gesture point cloud data, and the reconstructed features include the structural information between hand joint points and the local position reference information from points in the hand joint neighborhood to joint points, enriching the feature information of the gesture point cloud and effectively improving the robustness of gesture estimation. The overall network framework of this method is shown in Figure 1, which is divided into two modules: initial joint estimation guided by global features and hand joint refinement guided by local features.

### 3.1. Depth Map Preprocessing

In order to fully utilize the spatial depth information of the depth map, the depth map is converted into point cloud data, and the impact is reduced of viewpoint variations on the depth map representation of the same gesture, which would otherwise affect the estimation results. Principal component analysis (PCA) is used to process the gesture point cloud to obtain a rotation matrix. The gesture point cloud is then transformed into an Oriented Bounding Box (OBB) to ensure that the direction of the same gesture point cloud is consistent. In order to reduce the influence of different hand sizes on gesture estimation tasks due to different distances from the camera, the gesture 3D point cloud is normalized and centralized.

**Figure 2:**
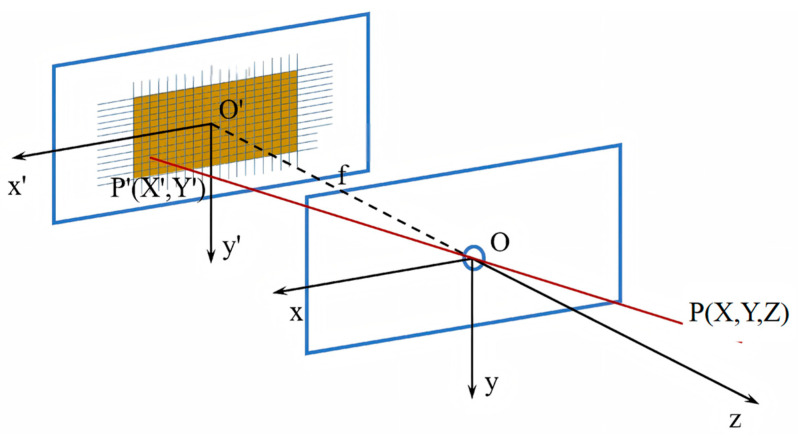
Pinhole camera model.

The bridge connecting the pixel coordinates and spatial physical coordinates of an image is the camera intrinsic parameter. The units of measurement for describing the size of scenery in the real world are meters, millimeters, etc. A camera presents a real-world scene in an image, with the size of the image measured in pixels. The pinhole camera model is shown in Figure 2. Assuming that the spatial point coordinates in the camera coordinate system *xyz* are *P* (*X*, *Y*, *Z*) projected onto the physical imaging plane *xO*′*y*′ at coordinates *P*′ (*X*′, *Y*′), and the camera focal length is *f*, we define *α* as the number of pixels per unit length in the *x*′-axis direction of the physical imaging plane, *β* as the number of pixels per unit length in the *y*′ direction of the imaging plane, and (*u*, *v*) as the pixel coordinates of *P*′ in the pixel plane.

The projection of objects in the pinhole camera model is inverted, and the camera generally reverses the image in the imaging plane to a positive image. The reversed point *P*″ (*X*″, *Y*″) has the following relationship according to geometric operations.

(1) $$X^{\prime \prime }=-X^{\prime }=f\frac{X}{Z},Y^{\prime \prime }=-Y^{\prime }=f\frac{Y}{Z}$$

Due to the fact that digital images captured by the camera are stored in pixels and the coordinate origin is located in the upper left corner of the image, *c_x_* and *c_y_* represent the horizontal and vertical pixel distances from the center of the image to the upper left corner. Combining Formula (1), the coordinate relationship between pixel coordinates and camera coordinates at point *P* can be obtained as follows:

(2) $$\left\{\begin{array}{c} u=\alpha X^{\prime \prime }+c_{x}=\alpha f\frac{X}{Z}+c_{x}\\ v=\beta Y^{\prime \prime }+c_{y}=\beta f\frac{Y}{Z}+c_{y} \end{array}\right.$$

Let *f_x_* = *αf* and *f_y_* = *βf*. Formula (2) can be written in matrix form as follows:

(3) $$\left[\begin{array}{c} u\\ v\\ 1 \end{array}\right]=\left[\begin{array}{ccc} f_{x} & 0 & c_{x}\\ 0 & f_{y} & c_{y}\\ 0 & 0 & 1 \end{array}\right]\frac{1}{Z}\left[\begin{array}{c} X\\ Y\\ Z \end{array}\right]$$

(4) $$K=\left[\begin{array}{ccc} f_{x} & 0 & c_{x}\\ 0 & f_{y} & c_{y}\\ 0 & 0 & 1 \end{array}\right]$$

Among them, *K* is the intrinsic parameter matrix of the camera. Converting a depth map to point cloud space essentially means mapping the pixel points of the depth map into physical spatial coordinate points. From the dataset, we can obtain the intrinsic parameters of the camera: *f_x_*, *f_y_*, *c_x_*, *c_y_*. Assuming that the pixel coordinates of the depth map are (*u*, *v*); the pixel value is *Z*, which represents the distance from the actual object to the camera (unit: mm); and the physical space point coordinates are (*X*, *Y*, *Z*), the transformation relationship is:

(5) $$\left\{\begin{array}{c} X=(u-c_{x})\times Z/f_{x}\\ Y=(v-c_{y})\times Z/f_{y} \end{array}\right.$$

**Figure 3:**
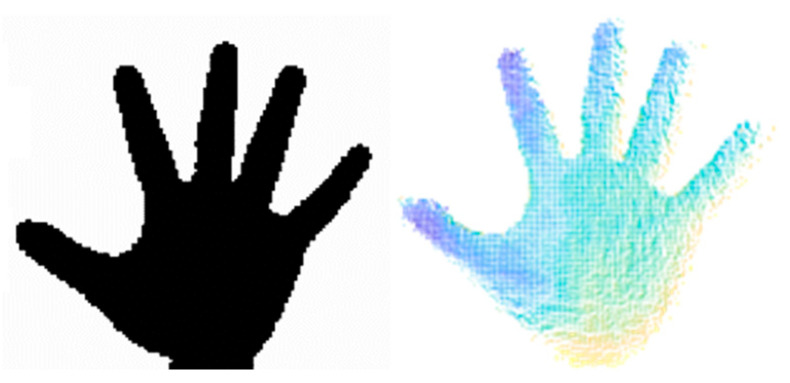
Conversion of depth map to point cloud.

The spatial coordinates (*X*, *Y*, *Z*) of each point in the depth map can be calculated based on the depth map pixels (*u*, *v*) and their pixel values. These points are the point cloud representations of the hand, as shown in Figure 3.

In actual interactive scenarios, human hand movements are frequent and the direction of the hand changes greatly. In order to avoid the problem of different depth map and hand area sizes for the same gesture due to different viewpoint and distances from the hand to the camera, this article converts the point cloud into an OBB with direction, which can include the hand point cloud and normalize it to reduce the differences in viewpoint and distance of the same gesture point cloud. Principal component analysis (PCA) is performed on the point cloud to calculate the direction of the OBB, where the coordinate axes *x*, *y*, and *z* of the OBB are aligned with the eigenvectors of the covariance matrix of the point cloud in descending order. Then the point cloud is moved to the center of the OBB coordinate system and normalized.

(6) $$p^{obb}={\left(R_{obb}^{cam}\right)}^{T}\cdot p^{cam}$$

(7) $$p^{nor}=(p^{obb}-p^{-obb})/L_{obb}$$

**Figure 4:**
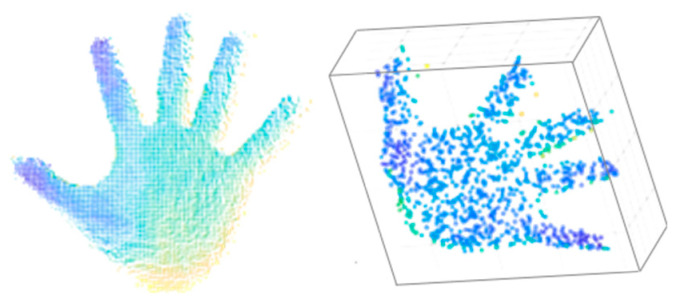
Normalized point cloud of OBB.

Among them, $R_{obb}^{cam}$ represents the rotation matrix of OBB in the camera coordinate system, *p^cam^* represents the coordinates of point *P* in the camera coordinate system, and *p^obb^* represents the coordinates of point *P* in the OBB coordinate system. In Formula (7), *p^-obb^* represents the center of the hand point cloud in the OBB coordinate system, *L_obb_* represents the maximum edge length of the OBB containing the point cloud in the OBB coordinate system, *p^nor^* represents the normalized coordinates of the OBB coordinate system, and the normalized point cloud coordinates are between [−0.5, 0.5]. The normalized point cloud of OBB is shown in Figure 4.

### 3.2. Global Feature Guided Initial Joint Estimation Module

**Figure 5:**
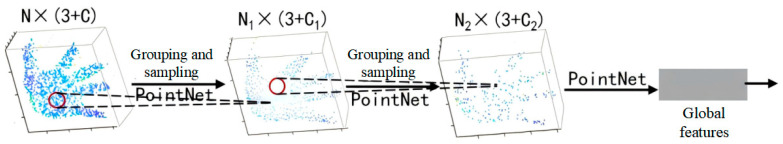
PointNet++ network structure.

The preprocessed hand point cloud is encoded using PointNet++ to extract global features. As shown in Figure 5, PointNet++ first uses FPS strategy to select grouped ball centers for the point cloud, and groups the point cloud using balls with a diameter of *R*. Then, PointNet is used to extract the global features of each point in the group, and the coordinates of the ball center are connected to the features of the local group region. This allows the PointNet++ structure, which applies FPS grouping and PointNet feature extraction at the hierarchical level, to obtain global features with richer local information compared to the global features obtained by the PointNet structure.

As shown in Figure 1, the encoder is a cascaded point set feature extraction layer. The point $p_{i}^{l}$ at the *l*-th layer (*l* ∈ {1, 2, *L*}) is obtained by downsampling the (*l* − 1)-th layer using FPS. The characteristics of $p_{i}^{l}$ are $f_{i}^{l}$:

(8) $$f_{i}^{l}=\underset{1\leq s\leq S}{Max}(h(cat(p_{i}^{l}-p_{i,s}^{l-1},f_{i,s}^{l-1})))$$

Among them, *Max* represents max pooling, and *S* is the number of intra group points grouped by the radius *r* of the sphere around the sampling point $p_{i}^{l}$ for the points in layer *l* − 1. *h* represents MLP, and *cat* represents connection operation. In this paper, the first layer of the PointNet++ encoder consists of each point feature connected by point coordinate $p_{i}^{l}$ and normal vector $f_{i}^{l}$. The last layer of feature extraction directly connects the point features and point coordinates of the previous layer and inputs them into the PointNet network to output a global feature $f^{L}$, then:

(9) $$f^{L}=\underset{1\leq i\leq N_{L-1}}{Max}(h(cat(p_{i}^{L-1},f_{i}^{L-1})))$$

**Figure 6:**
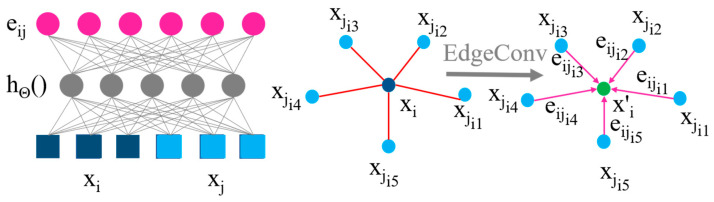
Edge convolution.

As shown in Figure 6, on the left, the edge features $e_{ij}$ for a pair of points $x_{i}$ and $x_{j}$ is computed, where $h_{\Theta }()$ represents a fully convolutional layer operation with shared weights. On the right is the EdgeConv operation, which aggregates the directed edge features of all neighboring points and edges of the point. Assuming there is a point cloud *X* with *n* points in dimension *F*, then $X=(x_{1},\ldots ,x_{n})$. Here, we assume *F* = 3, which means the point coordinates are $x_{i}=(x_{i},y_{i},z_{i})$.

Features such as color and normal vector can also be added. *G* (*v*, *ε*) is used to represent a directed graph in a point cloud, where *v* represents each point in the point cloud and *ε* represents a point-to-point directed edge. Given a directed graph of the nearest neighbors of a point, the edge feature is defined as $e_{ij}=h_{\Theta }(x_{i},x_{j})$, where $h_{\Theta }:R^{F}\times R^{F}\to R^{F^{\prime }}$ is a nonlinear mapping containing a set of learnable parameters *Θ*. EdgeConv is defined by applying a channel level symmetric aggregation operation □ (such as summation, maximization) on the edge features of all edges from each neighboring point to that point. The EdgeConv output of the *i*-th point is:

(10) $${x_{i}}^{\prime }=\underset{j:(i,j)\in \varepsilon }{□}h_{\Theta }(x_{i},x_{j})$$

EdgeConv essentially reconstructs an *F*-dimensional point feature into an *F*′-dimensional point feature that includes neighboring point features by combining the *F*-dimensional feature information of surrounding points. The choice of aggregation operation □ and $h_{\Theta }()$ mapping operation has a significant impact on the results and effects of EdgeConv. Aggregation operations include maximization, summation, averaging, etc., while mapping operations can be divided into several types.

The first method, similar to PointNet, only encodes global information and ignores local information:

(11) $$h_{\Theta }(x_{i},x_{i})={h^{\prime }}_{\Theta }(x_{i})$$

The second approach is to encode only local information and consider the shape as a collection of small pieces of information:

(12) $$h_{\Theta }(x_{i},x_{j})={h^{\prime }}_{\Theta }(x_{j}-x_{i})$$

The third method is to integrate local and global information, and this article uses this type of mapping operation:

(13) $$h_{\Theta }(x_{i},x_{j})={h^{\prime }}_{\Theta }(x_{i},x_{j}-x_{i})$$

The specific determination of characteristic parameters and the definition of parameter mapping functions also need to be set according to the specific application scenarios. Therefore, this article uses EdgeConv to reconstruct global features and takes into account the structural information between 2D hand skeleton joint points.

**Figure 7:**
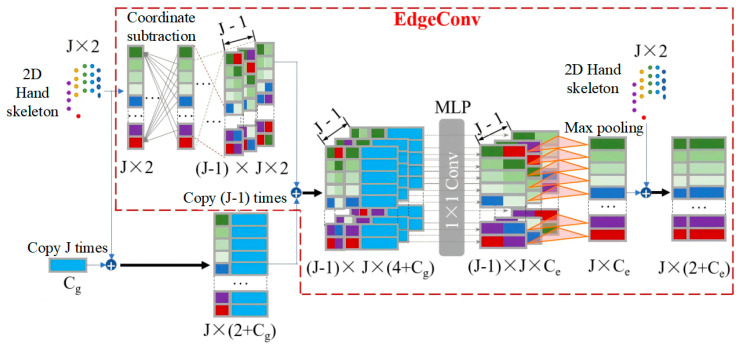
EdgeConv network structure for reconstructing global features.

**Figure 8:**
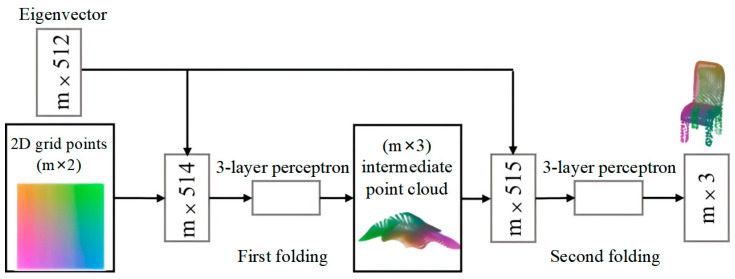
Folding decoding structure.

As shown in Figure 7, the global feature *f_g_* is replicated *J* times and concatenated with the 2D coordinates of each hand joint, forming joint-specific features, where *J* is the number of joints; the corresponding features of the joints are then copied *J* − 1 times and the coordinate difference *p_j_*-*p_i_* obtained by subtracting each other from the 2D hand skeleton plane coordinates and is connected to obtain the feature group of each hand joint relative to other joints. Here, *i* represents a certain joint in the 2D hand skeleton, and *j* represents any joint except for the *i*-th joint. Then, the feature groups of all joints are fed into an MLP to produce the directed edge features from each joint to every other joint. Finally, using max pooling operation, the directed edge features of each joint and other joints are aggregated to obtain new features that not only contain the original feature information of each joint, but also the structural information between joints. Guided by this new feature, folding decoding can obtain relatively accurate initial joint coordinates. Folding decoding is the operation of transforming points on a 2D grid into a 3D surface point cloud, which can achieve low reconstruction errors even for fine structures. The theoretical basis for folding decoding is that any 3D object surface can be transformed into a 2D plane through operations such as cutting, squeezing, and stretching. On the contrary, by performing certain folding operations to stick these 2D point samples back onto the object surface, these folding operations can initially be viewed as a 2D grid. The folding decoding structure is shown in Figure 8.

The folding operation is a three-layer perceptron, while the folding encoding framework consists of two layers of folding operations. The folding operation can be described as a virtual “force” that acts on a 2D grid to simulate the learning process of a folding network, causing the 2D grid to stick to a spatial surface. The folding operation can be seen as a 2D to 3D nonlinear occultation. Assuming that the grid has *m* points, we copy the features (codewords) obtained from the encoder *m* times and connect them to the grid coordinates as the input features of the folding operation. The input features of the folding operation not only include the 2D grid points, but also the parameter codewords that guide the folding operation. An MLP is close to a nonlinear mapping function. Using MLP, complex folding operations can be performed on a 2D grid, while high-dimensional codewords store the “force” that guides folding. This high-dimensional input makes folding operations more diverse, so folding operations can fold a 2D grid onto any shape of surface. Two layers of folding operation can accurately fold a 2D grid to any surface, so using more layers of folding will increase the network model parameters and affect computational efficiency. The edge feature *e_ij_* is:

(14) $$e_{ij}=h_{\Theta }(x_{i},x_{j})=h(cat(p_{j}-p_{i},p_{i},f_{g})$$

By using Formula (14), the directed edge from the *i*-th joint to the *j*-th joint among the other joints is calculated. Then, a max pooling operation can be applied to the directed edge features of the *i*-th joint and all other joints to obtain a feature $f_{e}^{i}$ that contains the *i*-th joint feature and structural information between the *i*-th joint and other joints. This feature is used to guide the folding decoding structure to obtain the initial 3D spatial coordinates of the *i*-th joint.

(15) $$f_{e}^{i}=\underset{1\leq j\leq J-1}{Max}(h(cat(p_{j}-p_{i},p_{i},f_{g})))$$

**Figure 9:**
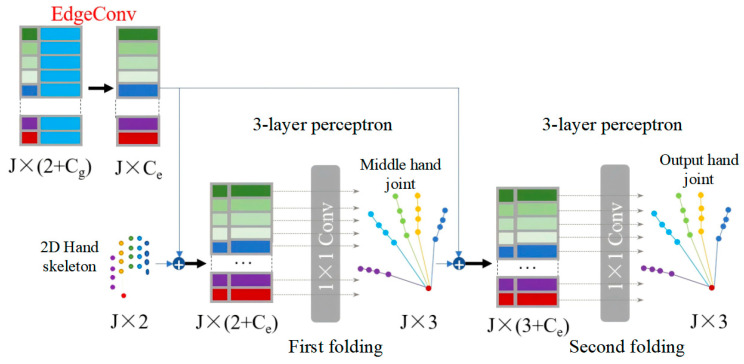
Estimating the folding decoding structure of the initial hand joint.

The guiding features of all joint folding decoders in the 2D skeleton is calculated using Formula (15). The decoding structure for estimating the initial hand joint coordinates is shown in Figure 9.

The difference between the folding structure in Figure 7 and Figure 9 is that the 2D hand skeleton is folded into 3D space to obtain the spatial coordinates of the hand joints in this paper. Assuming that the coordinates of the *i*-th joint in the 2D hand skeleton are $skel_{i}$, the coordinates output by the first level folding decoding are $e_{i}$, the initial hand joint coordinates output by the second folding decoding are $j_{i}^{o}$, and the initial hand joint coordinates are:

(16) $$\begin{array}{l} j_{i}^{o}=h_{p}(cat(e_{i},f_{e}^{i})),\\ e_{i}=h_{e}(cat(skel_{i},f_{e}^{i})) \end{array}$$

**Figure 10:**
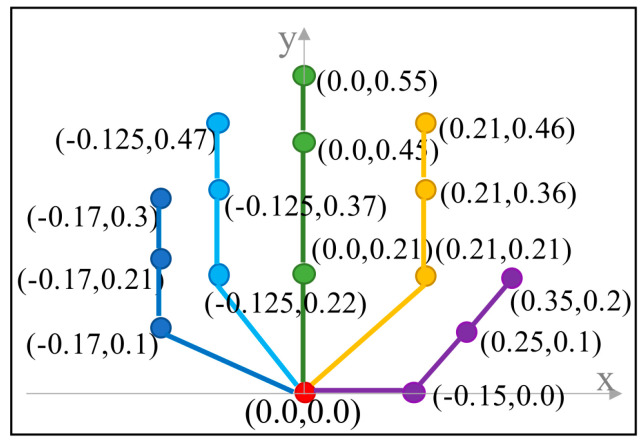
2D hand skeleton of ICVL dataset.

Among them, *h_e_* () and *h_p_* () respectively represent the three-layer perceptron for the first folding and the three-layer perceptron for the second folding. The 2D hand skeleton randomly selects sample data on the training set of the dataset, connects the label coordinates of adjacent joint points of the samples to calculate the finger joint length, and calculates the average length of all samples. By recording the average length of all knuckles in this way and disassembling them onto a plane, a 2D hand skeleton can be obtained. The 2D hand skeleton of the ICVL dataset is shown in Figure 10.

### 3.3. Local Feature Guided Joint Refinement Module

**Figure 11:**
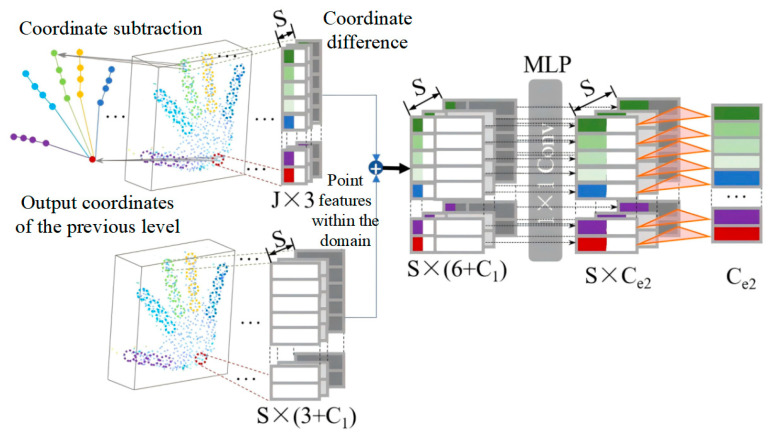
The architecture of EdgeConv firstly constructing local features.

The second stage is to locally modify the hand joint coordinates output by the previous level. EdgeConv is used for two rounds of local joint feature construction. The first round considers the position reference information between the local neighborhood points centered on the joint points output by the previous level, and the structure of the first round of local feature construction is shown in Figure 11.

As shown in Figure 11, the hand off node coordinates output from the previous module and the layer l gesture point cloud features of the PointNet++ extraction module are taken as the input of EdgeConv’s first construction of local features, and the hand joint coordinates output from the previous module are taken as the center to sample the gesture point cloud features in groups. The features of each point within the group and the coordinate difference between each point and its center are connected, and inputted into an MLP; the directed edge features are output from the points within the group to their center, and then the max pooling operation is used to obtain the first constructed local features representing each joint. The first constructed local feature $f_{e2}^{i}$ for the *i*-th joint is:

(17) $$f_{e2}^{i}=\underset{1\leq s\leq S}{Max}(h(cat(p_{s}^{i}-p_{c}^{i},f_{s}^{i})))$$

**Figure 12:**
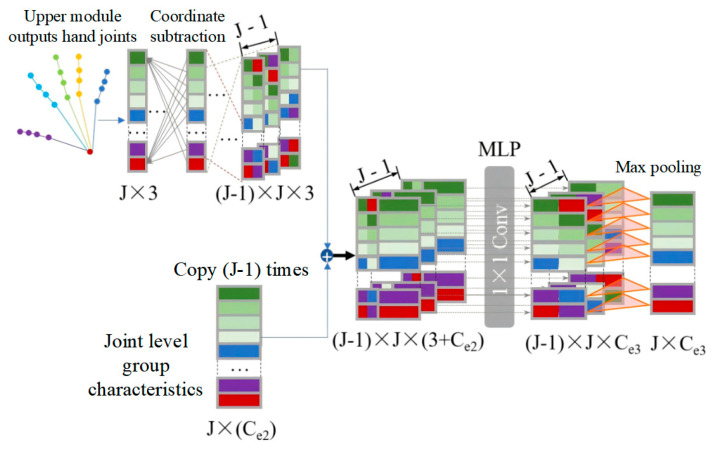
The architecture of EdgeConv secondly constructing local features.

Among them, $p_{s}^{i}-p_{c}^{i}$ represents the coordinate difference between each point in the *i*-th group and its center after grouping, and $f_{s}^{i}$ represents the characteristics of each point in the group. The second construction considers the structural information between hand joint points, and the structure of the second local feature construction is shown in Figure 12.

As shown in Figure 12, this paper takes the manual node coordinates output from the previous module and the local features constructed for the first time by EdgeConv as the input of the local feature structure constructed for the second time by EdgeConv. The local features constructed for the first time are copied *J* − 1 times and the two coordinate differences of the hand joint coordinates output from the previous module are connected, and then fed into an MLP to output the directed edge features of other joints to a joint. Max pooling is applied to aggregate all other joint points to the directed edge features of the joint point to obtain the local features of each joint constructed for the second time. The local feature $f_{e3}^{i}$ of the *i*-th joint constructed for the second time includes:

(18) $$f_{e3}^{i}=\underset{1\leq j\leq J-1}{Max}(h(cat(p_{j}-p_{i},f_{e2}^{i})))$$

**Figure 13:**
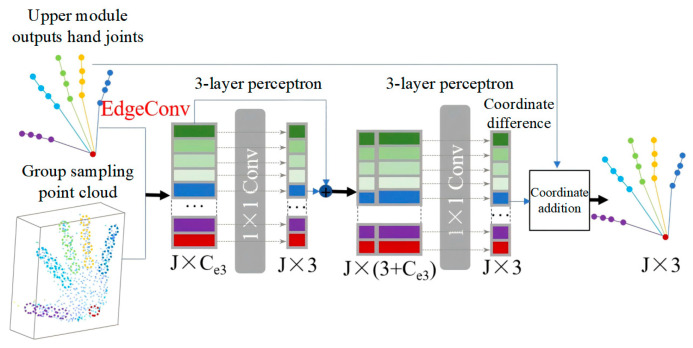
Local folding decoder architecture.

Among them, $p_{j}-p_{i}$ represents the coordinate difference between the *i*-th hand joint output by the upper level and the *j*-th hand joint among the other joints. By inputting the constructed local joint features into a local folding decoding structure, the coordinate difference between the upper level joint coordinates and the real label coordinates can be obtained, thereby achieving correction of the upper level output hand joint coordinates and improving the estimation accuracy of hand joints. The folding decoding structure is similar to the fully absolute folding decoding structure, but the difference is that the global folding decoding objective is to fold the 2D hand skeleton joints to the 3D space hand joint positions, while the local folding decoding objective is to obtain the difference in hand joint coordinates output by the previous level structure compared to the real label positions. The partial folding structure is shown in Figure 13.

As shown in Figure 13, the input of the local folding decoder is the output hand joint coordinates of the previous module and the characteristics of the layer l special gesture point cloud. Group sampling is performed on the *l*-th layer point cloud features of the PointNet++ feature extraction module, with the upper level output joint as the center. Guiding features are constructed using EdgeConv, and then input into the folding decoding module to obtain coordinate differences. The calculation for the *i*-th joint correction hand is:

(19) $$j_{i}^{k}=h_{p}(cat(h_{e}(f_{e3}^{i}),f_{e3}^{i}))+j_{i}^{k-1}$$

Among them, $j_{i}^{k-1}$ and $j_{i}^{k}$ represent the coordinates of the previous level and the locally corrected coordinates, $f_{e3}^{i}$ represents the local features grouped around the joint points of the previous level, and $h_{e}()$ and $h_{p}()$ represent the first and second folding operations, respectively.

### 3.4. Loss Function

The loss function used in this article is the *smooth L*1 loss function, which is less sensitive to outliers compared to the *L*2 loss function. The training process is relatively stable, and the *smooth L*1 loss function is:

(20) $$L1_{smoot}(x)=\left\{\begin{array}{c} 0.5\left|x\right|,\\ \left|x\right|-0.005, \end{array}\begin{array}{c} \left|x\right|<0.01\\ \left|x\right|>0.01 \end{array}\right.$$

Due to the fact that both the global joint roughness estimation module and the local joint refinement module in the network output coordinates, the loss function needs to supervise the outputs of all modules. Then we have:

(21) $$Loss=\sum_{i=1}^{J}L1_{smooth}(j_{i}^{o}-j_{i}^{*})+\sum_{k=1}^{K}\sum_{i=1}^{J}L1_{smooth}(j_{i}^{k}-j_{i}^{*})$$

Among them, $j_{i}^{*}$ represents the true label coordinates of the *i*-th joint point, and *K* represents the number of fast local folds.

## 4. Experimental Results and Analysis

We train and test our method on 3D point cloud datasets ICVL, NYU, and MSRA, compare the accuracy of our proposed method with existing mainstream point cloud gesture estimation algorithms, and analyze the performance of our proposed method in depth. The experimental hardware CPU used in this article is Intel (R) Xeon (R) Gold 6330, operating at 2.00 GHz, and a PC server with NVIDIA RTX 4090 24 GB GPU and 256 GB memory. The operating system is Ubuntu 22.04, on which we create a virtual environment with Python 3.10 using Anaconda3. The network framework is built and trained with PyTorch 2.1 in this environment, and PyCharm Professional Edition is used as the code editor. We choose the Adam optimizer with *β*1 = 0.5, *β*2 = 0.99, and learning rate *α* = 0.001.

We sample 1024 points from the gesture 3D point cloud as input for the network architecture. The batch size is set to 32. Since many MLPs in the network output coordinates or coordinate differences, the ReLU activation function is chosen for the MLPs. To avoid overfitting, data is enhanced through rotation, scaling, and deformation. The maximum number of training sessions for each dataset is set to 400 epochs for the ICVL dataset, 200 epochs for the NYU dataset, and 80 epochs for the MSRA dataset. The selected hand joint coordinates are used throughout the model training process, and the skeleton topology, number of joint points, and relative positions will not change. Only the network model parameters are iteratively optimized [29]. All EdgeConv layers are uniformly set to *k* = 20, and each layer of the network will reconstruct the topology to ensure the reproducibility of the experimental results.

### 4.1. Dataset

Dataset is crucial for supervised deep learning tasks. For gesture pose estimation, large-scale, high-precision, and highly applicable gesture pose datasets can not only provide accurate performance testing and method evaluation, but also promote the development of gesture pose estimation research. Among them, ICVL, NYU, and MSRA are the most widely used 3D point cloud gesture estimation datasets, as shown in Table 1 for specific information.

**Figure 14:**
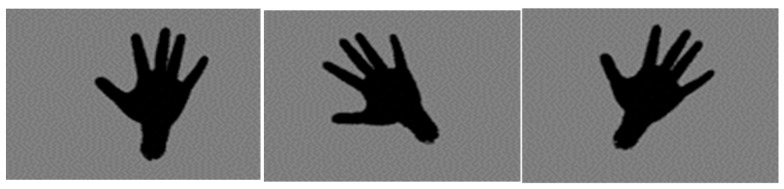
Depth maps of ICVL with the same gesture but different viewpoints.

As shown in Figure 14, ICVL [30] was provided by the Vision Laboratory at Imperial College London, and the training set consisted of a total of 180 k with 320 × 240 depth frames collected from 15 different viewpoints by 10 different subjects for 26 different gestures. The test set consists of depth maps of approximately 800 frames each for two different subjects. The dataset uses a 16-joint model, and the coordinates of the marked joints exist in the corresponding txt document.

**Figure 15:**
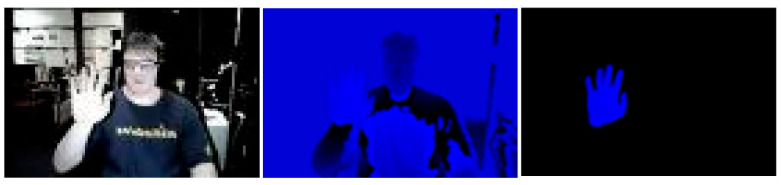
NYU color map, depth map, and synthetic hand depth map.

As shown in Figure 15, NYU [31] is provided by New York University. The training set contains 72,757 frames from a single subject captured from three viewpoints, each with corresponding depth maps, color images, and synthetic hand-only depth maps of size 640 × 480. The training set also includes 8252 frames of depth maps, color images, and synthetic hand regions from two subjects. The dataset uses a 36-joint model, and the labeled joint coordinates are in the Joint_data.mat file.

**Figure 16:**
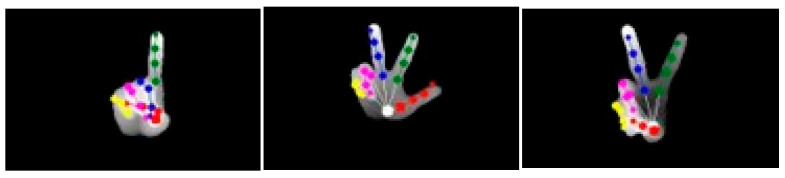
Rendering of depth and realistic labels for different gestures in MSRA.

As shown in Figure 16, MSRA [32] contains the right hands of nine different subjects, and gestures for each person, each gesture having approximately 500 frames of a 320 × 240 depth map. The dataset uses a 21-joint model, and the marked joint coordinates are stored in the joint.txt file. JPG format files are only used for visualizing depth maps and joint positions. The depth pixel values of each dataset are included in the bin file.

### 4.2. Preprocessing and Visualization Results

**Figure 17:**
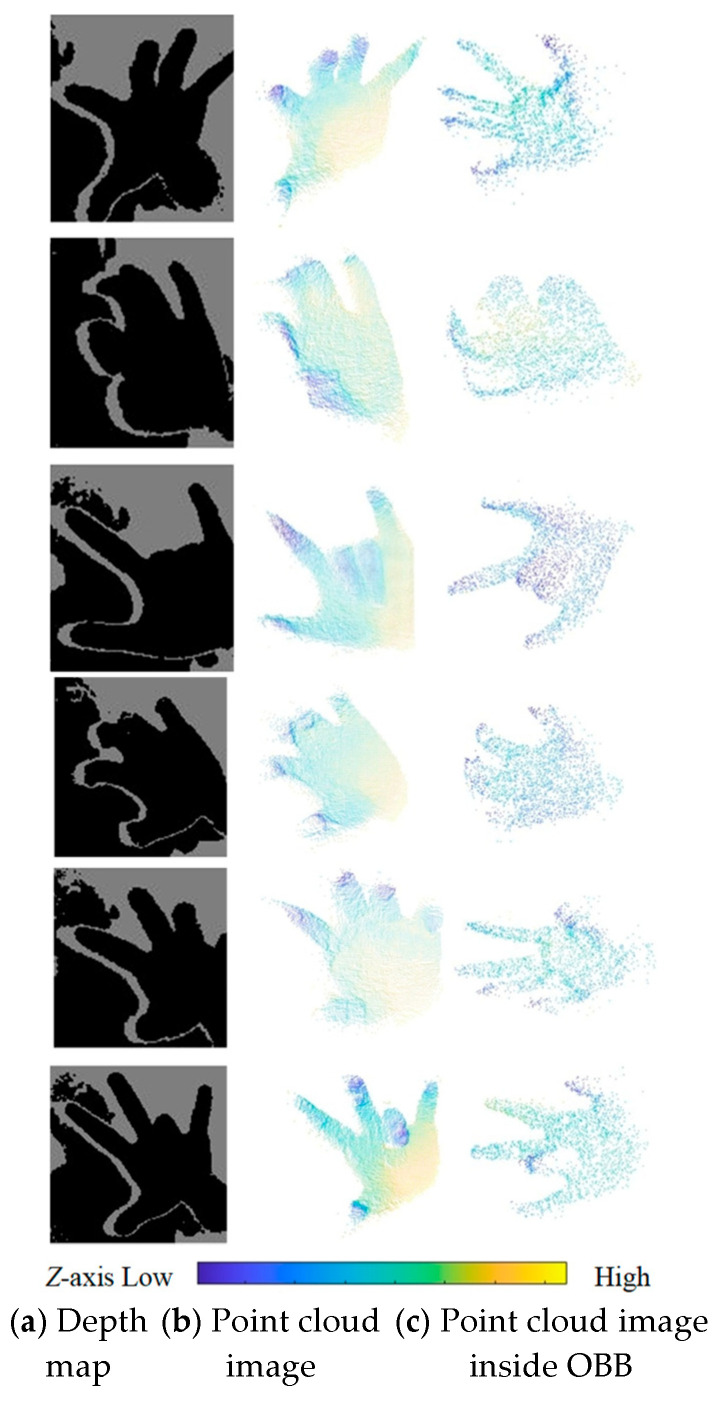
Depth map converted into point cloud data processing result visualization.

Converting depth maps into point cloud data can reduce the impact of different perspectives on gesture estimation results for the same depth map. The visualization results of converting the depth map into point cloud data processing in this article are shown in Figure 17, which is a randomly selected depth map from the ICVL dataset test set. Hand region detection is performed based on the depth map threshold, and the hand region depth map is converted into a point cloud map. We use the Farthest Point Sampling (FPS) strategy to downsample the gesture point cloud map. PCA is performed processing on the downsampled gesture point cloud to transform it into the OBB coordinate system, followed by normalization and centralization. Figure 17a shows the depth map, Figure 17b shows the original 3D point cloud data converted from the depth map, and Figure 17c shows the 3D point cloud data after normalization and centralization in the OBB coordinate system. All point cloud visualizations in Figure 17 are visual views along the positive Z-axis starting from the origin of their respective coordinate systems.

**Figure 18:**
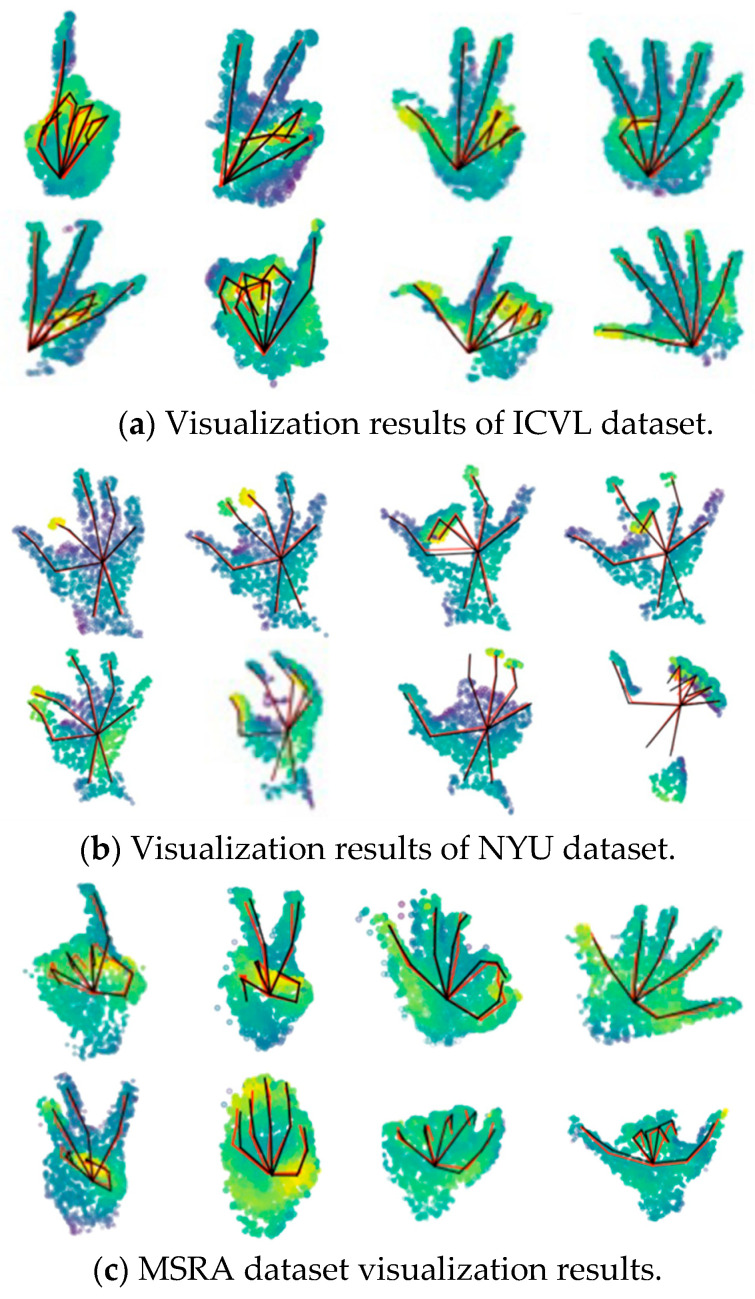
Visualization results of gesture estimation results.

We convert the test set depth maps of the ICVL, NYU, and MSRA datasets into point clouds, and visualize the real labeled joint points and predicted hand joint points on the gesture point cloud as shown in Figure 18.

As shown in Figure 18, the black line represents the connection of real label joint point coordinates, and the red line represents the connection of gesture joint point coordinates estimated by the method proposed in this paper. The visualization results are for the ICVL, NYU, and MSRA datasets, respectively. From Figure 18, it can be seen that the overlap between the red and black lines is high, indicating that the method proposed in this paper has high accuracy in testing results on three popular 3D point cloud datasets.

### 4.3. Experimental Results of Comparative Experiments

**Figure 19:**
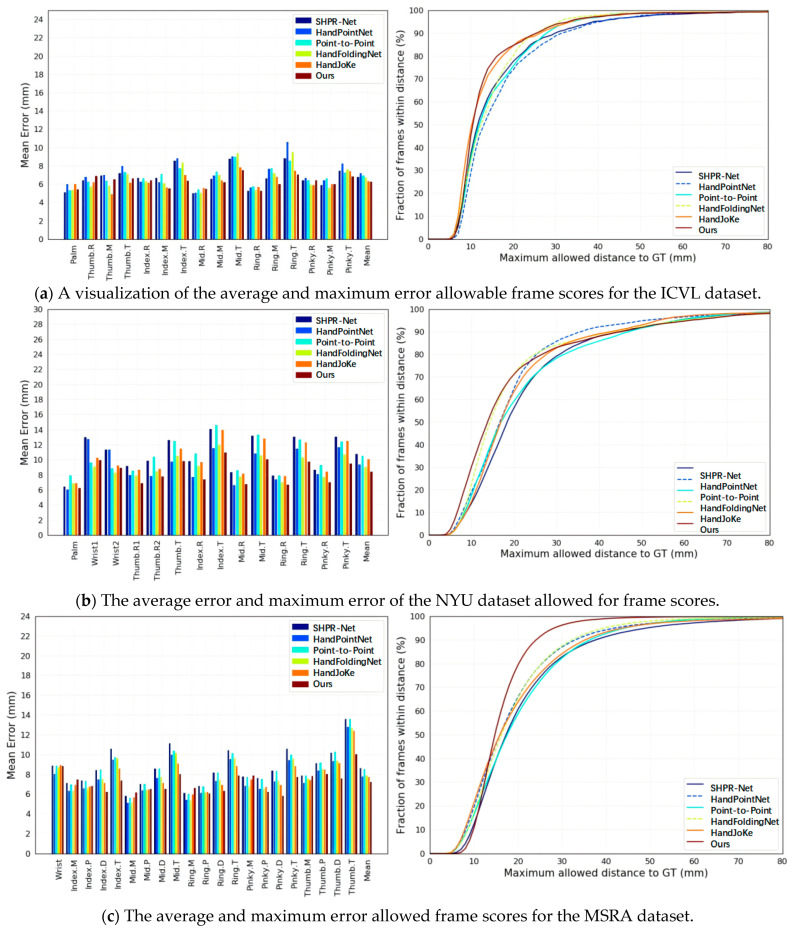
Average error histogram and fraction of frames within maximum allowed error distance curve.

We conduct comparative experiments between our method and the existing mainstream 3D point cloud methods, including SHPR-Net [26], HandPointNet [25], Point-to-Point [28], HandFoldingNet [9], and HandJoKe [24]. The indicators are divided into two aspects: average error and maximum allowable error frame score. (1) The average error in this article consists of two parts: the average error of each joint on the test set and the average error of all joints on the test set. (2) The maximum allowable error frame score is the measure of the success rate of the gesture estimation task in the test set when the maximum estimation error of all joints is less than a measurement value, which is considered successful. We plot the curves of the maximum allowable error frame fraction for both our method and the compared methods under varying tolerance values, and we compare and analyze the performance of our method against the other algorithms. The maximum allowable error frame score is a very strict evaluation indicator for measuring the success of gesture estimation tasks based on the maximum error. The average error histogram and maximum allowable error frame score curve of gesture estimation results are shown in Figure 19.

As shown in Figure 19, from top to bottom are the average error histograms and maximum allowable error frame score curves of the ICVL, NYU, and MSRA datasets, respectively. The left figure shows the histograms of the average gesture estimation error and total average error for each node on the test set using different methods. Table 2 presents the statistical analysis of the total average error for different methods. Both the average error histogram in the left figure of Figure 19 and Table 2 indicate that our method outperforms other methods in terms of estimation accuracy. The right figure in Figure 19 is the curve of the maximum allowable error frame score. The horizontal axis represents the maximum error boundary value for measuring the success of gesture estimation, and the vertical axis represents the success rate of the estimation task, that is, the proportion of successful gesture estimation images in the test set to the total number of images in the test set in the maximum error boundary value for measuring success on the horizontal axis. Our method is significantly superior to other methods on the ICVL dataset. On the NYU dataset, our method outperforms other methods significantly before a maximum error of 25 mm, but after 25 mm, due to the large error, it lacks practical application significance. On the MSRA dataset, it can be seen that within a very small range of measurement values, the curve of our method reaches the vertex first, indicating that our method has better stability than other methods.

### 4.4. Experimental Results of Complex Scenarios

**Figure 20:**
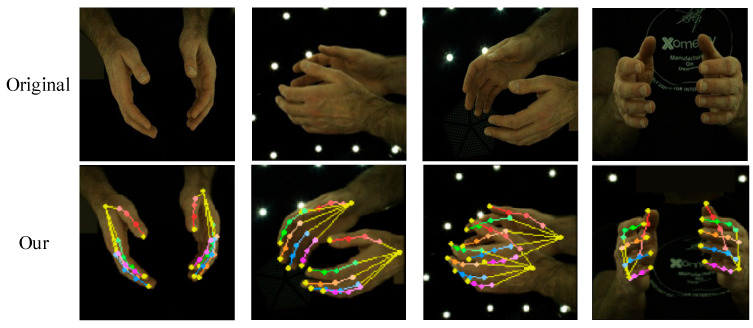
Partial visualization results of InterHand2.6M dataset.

To further analyze the performance and generalization of the proposed method in complex scenes such as occlusion, rapid motion, and skin color similarity, experimental analysis was conducted on the InterHand2.6M dataset, which is a large-scale real RGB image dataset composed of complex scene hand poses of single hand and interactive hand sequences. The visualization results of the experimental results are shown in Figure 20. The method proposed in this paper has more accurate gesture estimation, clearer differentiation of different finger joint positions, better ability to distinguish between two hands, and more accurate joint position prediction. These results fully demonstrate that the method proposed in this paper has better gesture estimation performance in dealing with complex problems such as occlusion and rapid motion in hand pose estimation.

Given that fingertip joints often exhibit significant errors, each joint is subdivided to further evaluate and analyze the performance of our method. For the InterHand2.6M dataset, this paper uses three metrics to evaluate gesture prediction results: APh (Average Precision of Dominant Hand Estimation), Mean Relative Root Position Error (MRRPE), and Mean Per Joint Position Error (MPJPE). Specifically:

(22) $${\mathrm{AP}}_{h}=\frac{m}{n}$$

(23) $$MPJPE=\frac{1}{n}\sqrt{\sum_{1}^{n}{\left(\hat{p}-p\right)}^{2}}$$

(24) $$MRRPE=\frac{1}{n}\sqrt{\sum_{1}^{n}\left(z^{R-L^{*}}-z^{R-L^{*}}\right)}$$

Among them, m is to predict the correct number of images for gesture categories, n is the number of all gesture images, $z^{R\to L^{*}}$ is the depth of the real right hand relative to the left hand, $\hat{p}$ represents the predicted joint position, and p represents the real joint position.

**Figure 21:**
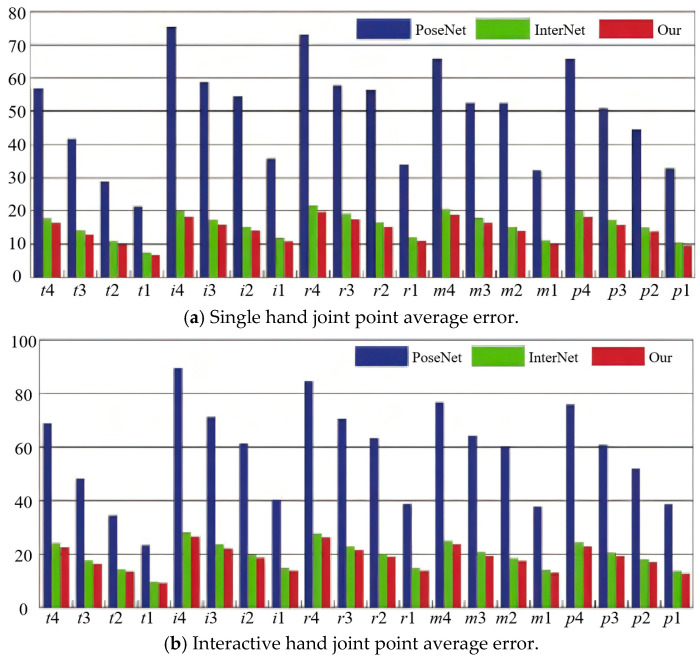
Average error of interactive hand joint points.

On the InterHand2.6M dataset, in order to further validate the performance of the proposed method in terms of average error of hand nodes during interaction, different methods were used to test single hand and interactive hand images, and the average value of left and right hand joint points was taken as the error of each joint point. The experiment was conducted using PoseNet, InterNet, and the method proposed in this paper. The results are shown in Figure 21, where 1, 2, 3, and 4 correspond to the joint points from the base of the fingers to the fingertips, and *t*, *i*, *m*, *r*, and *p* represent the thumb, index finger, middle finger, ring finger, and little finger, respectively. It can be seen that predicting joints near the palm is more difficult than predicting joints near the fingertips. The average error rate of our method on different finger joints is lower than other comparison methods.

We compare the method of this article with PoseNet [33], InterNet [34], DIGIT [35], Keypoint [36], HDRNet [37], GroupPoseNet [38], Handformer2T [39], mmWave-HGR [40], and BCPoseNet [3]. The latest method was used for comparative experiments, and the results are shown in Table 3, where bolding is the optimal value.

As shown in Table 3, the method proposed in this paper performs excellently in terms of APh and average error of the root joints of both hands, with values of 99.05% and 10.32 mm, respectively, indicating that the proposed method has higher robustness in some scenarios of two-hand interaction and occlusion. In terms of the average error of single-handed root nodes and joint nodes, this paper’s method performs well, with values of 8.92 mm and 26.38 mm, respectively. This is mainly due to the effective modeling of the overall structure relationship of the hand using a global feature guidance module, which significantly improves the accuracy of root node localization relying on global information. In addition, the local feature fusion module enhances the feature discrimination ability in the case of occlusion between the two hands, reducing the estimation error in the scene of two-hand interaction. The Keypoint method decouples keypoint localization and joint matching, taking into account both hand and object tasks. It has a small number of parameters and robust occlusion, but the training process is cumbersome. GroupPoseNet uses grouped convolution to isolate the left and right hands, but there are issues with redundant feature branches and weak differentiation between left and right hands. HDR uses image restoration to erase interfering hands, transforming two-handed tasks into one-handed recognition with outstanding accuracy. However, multi-stage pipeline inference is slow, and image artifacts are prone to occur during restoration. HDRNet builds a lightweight gesture framework based on depth maps, relying on geometric constraints to ensure accuracy, but only supports deep devices and cannot handle both hands. MmWave HGR relies on radar to achieve non-visual privacy recognition, significantly reducing false alarms based on the hand lift trigger mechanism, but can only recognize fixed gestures and has a high hardware threshold. However, although the method proposed in this article has a significant improvement in accuracy compared to Handformer 2T, it is inferior in terms of the average root node error and joint point error of a single hand compared to Handformer 2T. This is because the pose query enhancement module iteratively corrects occlusion joint errors, has strong occlusion robustness, and is compatible with single/dual hand images, without the need for special branches for single hands. In contrast, the Handformer 2T pure regression method does not have an explicit keypoint localization process, only outputs joint coordinates, does not support hand mesh and object pose prediction, and extremely heavily overlaps with both hands, making it easy to confuse left and right hand boundaries globally. Therefore, this method optimizes the joint correction module guided by local features, further improving the ability to capture the detailed structure of gesture estimation, with good accuracy and generalization.

### 4.5. Ablation Experiment

To verify the effectiveness of the global EdgeConv, the first step local feature initial joint estimation module, and the second step local feature joint correction module for gesture pose estimation, ablation experiments were conducted on different modules. Due to the fact that this method is a 3D-point-cloud-based approach, all the experiments were trained and tested on the MSRA dataset for fair comparison. The results are shown in Table 4.

As shown in Table 4, the method proposed in this paper achieves the best results after processing with global EdgeConv, first step local features, and second step local features. This indicates that the 3D hand pose estimation algorithm has achieved optimal overall performance under the collaborative action of the three modules. This fully validates the effectiveness of each module and provides a more accurate and stable solution for hand pose estimation. In addition, due to the different complexity of the dataset and its scenario-oriented approach used in this article, the results in Table 1 and Table 4 differ.

### 4.6. Real-Time Analysis

**Figure 22:**
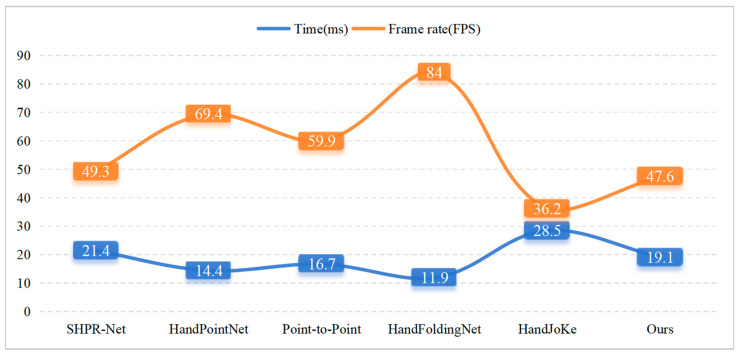
Real-time comparison visualization results.

On the ICVL, NYU, and MSRA datasets, we conduct a real-time comparative analysis between our method and the mainstream 3D point cloud methods, including SHPR-Net [26], HandPointNet [25], Point-to-Point [28], HandFoldingNet [9], and HandJoKe [24]. The results are averaged over nine repeated experiments, and the real-time comparison results are shown in Table 5, with visualizations presented in Figure 22.

As shown in Figure 22, the average computation time for one hand pose estimation in this method is about 19.1 ms, and the improved average computation time is about 47.6 FPS, basically achieving a balance between accuracy and real-time performance. The HandJoKe method is a multimodal gesture estimation work for point clouds and depth maps. The core innovation is to guide the Transformer to filter point cloud noise and background redundant points through joint priors, but its real-time performance is poor. Although the real-time performance of the HandFolding Net method can reach up to 84 FPS, it relies on shallow networks for high frame rates, resulting in relatively low gesture estimation accuracy. In the future, this article can further improve the inference speed to real-time level through optimization methods such as model quantization and operator fusion, achieving a better balance between accuracy and speed.

**Figure 23:**
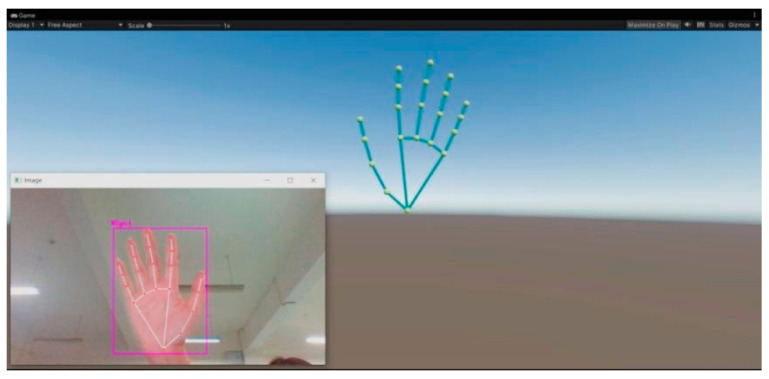
Results of mapping real hand joint points to virtual hand.

**Figure 24:**
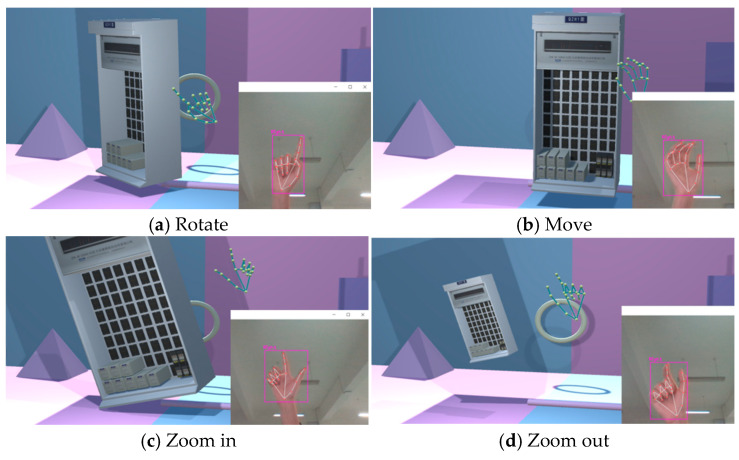
Gesture estimation interactive application.

To further analyze the usability and real-time performance of the method proposed in this article in practical application scenarios, a virtual hand model was constructed in the Unity3D 5.2 environment, and the algorithm-estimated hand joint coordinates were mapped in real-time to the model, thereby achieving visual presentation and immersive control of gesture actions. The mapping effect between the real hand joint points and the virtual hand model is shown in Figure 23. As shown in Figure 24, by recognizing fingertip rotation gestures to control the rotation of the model, the system will display real-time mapping results of camera-captured gesture actions. By recognizing fingertip rotation gestures to control the rotation of the model, the shape and structure of the 3D model can be clearly observed from multiple angles, and model movement and scaling can also be performed. In the actual interactive application stage, it was found that the method proposed in this paper can meet the real-time requirements of practical interactive applications.

## 5. Discussion

To consider the structural information between joint points and the mutual reference information between local neighboring points, this paper introduces the EdgeConv principle in graph convolution and proposes a 3D point cloud gesture estimation method based on EdgeConv to reconstruct joint features for accurate estimation of hand node information. In response to the common industry problems of insufficient joint topology association mining, shallow detail feature loss, low accuracy of complex gesture pose restoration, and poor robustness in occluded scenes in traditional 3D point cloud gesture pose estimation methods based on convolutional neural networks and Transformers, EdgeConv is utilized to adapt the topological structure of hand skeleton and reconstruct and optimize hand joint features. Based on the comparative and ablation experiments of the 3D point cloud gesture dataset, it can be concluded that this method can effectively capture the spatial correlation features between the joints of the hand, compensate for the weak global modeling ability of ordinary convolution and its inability to adapt to irregular topological structures of the hand, significantly improve the accuracy and real-time performance of 2D and 3D gesture pose estimation under monocular vision, and verify the effectiveness and feasibility of the EdgeConv joint feature reconstruction scheme in 3D point cloud gesture estimation tasks.

In gesture interaction, human hand movements are frequent and the direction changes greatly, and hand joints are prone to self-occlusion. Compared with traditional gesture estimation methods, this paper mainly focuses on optimizing the logic of 3D point cloud feature modeling. In contrast to simple graph convolution and standard convolution 3D point cloud gesture estimation models, this paper’s method has stronger adaptability to complex gesture states such as overlapping fingers, slight occlusion, and angular deviation, and can accurately restore the pose changes of continuous dynamic gestures, effectively reducing gesture joint estimation errors [29]. However, traditional convolution methods can only extract planar features based on local pixel windows, ignoring the inherent topological constraints and spatial dependencies between hand joints, which can easily lead to problems such as joint coordinate displacement, finger pose confusion, and failure to recognize subtle gesture differences. Although Transformer algorithms have global modeling capabilities, they suffer from the drawbacks of large parameter count, high computational redundancy, and poor real-time performance, making it difficult to adapt to real-time gesture interaction scenarios in embedded terminals [41]. The EdgeConv module introduced in this article is based on the modeling unit of hand joint points and bone edges, which conforms to the physiological structural characteristics of the hand and reconstructs discrete joint features in a targeted manner. It transforms isolated joint pixel features into structured features with topological correlations, while preserving the subtle posture details of the hand.

Although the method proposed in this paper has achieved good results in 3D point cloud gesture estimation tasks, further analysis based on experimental data and actual natural interaction scenarios shows that this method still has certain limitations. We will mainly focus on conducting in-depth research on real-time operational efficiency and dynamic interaction performance. Firstly, the model lacks robustness in extremely complex environments. When there is extensive occlusion, overlapping of multiple hands, overexposure of strong light, or interference from dark shadows on the hand, a large number of effective features of key joint points in the hand will be lost, making it difficult for the EdgeConv module to complete the complete topology reconstruction, resulting in an increase in joint coordinate prediction error and significant distortion of gesture posture. Secondly, the focus of this article is to optimize the extraction of topological features in hand space, without building an inter-frame temporal feature capture module. Although the inference latency of the model in natural human–computer interaction scenarios has been quantitatively analyzed through runtime performance testing, it has been verified that the proposed method meets real-time interaction requirements. However, the temporal modeling ability of the proposed model is difficult to adapt to high-speed dynamic gestures. In the face of rapid and continuous dynamic gestures in natural interaction, the algorithm cannot accurately capture the attitude evolution between adjacent frames, resulting in insufficient stability in continuous gesture tracking and attitude estimation. Finally, the current model only relies on publicly available standard datasets for training. Although further generalization experiments were conducted on the InterHand2.6M dataset in this paper, the adaptation effect of the model may decline to a certain extent for custom gesture samples with diverse skin tones, differentiated hand sizes, and cluttered backgrounds in real environments. Therefore, the cross-scenario generalization performance of the model in this paper needs to be further improved and optimized based on actual application scenarios and requirements.

In summary, the method proposed in this paper can be iteratively optimized from three dimensions in the future. Firstly, by introducing attention mechanism and occlusion repair module, the inference and completion of missing joint features are achieved for hand occlusion scenes, enhancing the anti-interference ability of the model in complex interference scenes and improving the accuracy of extreme scene gesture estimation. Secondly, by integrating temporal convolutional networks and recurrent neural networks, a spatiotemporal dual branch feature modeling structure is constructed to synchronously capture the spatial topological features of gestures and the temporal variation features between frames, adapt to continuous dynamic gesture estimation tasks, and expand the dynamic scene adaptation capability of the model [42]. Thirdly, by constructing a self-built 3D point cloud gesture dataset with multiple scenes and diverse backgrounds; expanding gesture samples under complex backgrounds, multi-hand interactions, and extreme lighting conditions; and combining data augmentation algorithms to optimize the model training process, the adaptability of the model in practical application scenarios can be improved [43]. At the same time, lightweight pruning and operator optimization can be carried out on the model in the future to further reduce the number of model parameters and inference delay, adapt to the real-time lightweight natural gesture interaction needs of mobile devices and embedded devices, and promote the widespread application of 3D point cloud gesture estimation technology in human–computer interaction, intelligent wearables and other scenarios.

## 6. Conclusions

This article focuses on the frequent hand movements and significant changes in movement direction of users, which can cause self-occlusion of hand joints and make gesture estimation difficult. It is difficult for the accuracy of gesture estimation to meet the needs of natural gesture interaction applications. This article proposes a 3D point cloud gesture estimation method based on EdgeConv reconstruction of joint features to improve the robustness of gesture estimation. By introducing EdgeConv for feature reconstruction of joint point features, and using EdgeConv to reconstruct gesture 3D point cloud features in the initial estimation stage of hand joint nodes, the information between points and their neighboring points is taken into account when reconstructing point features based on EdgeConv. In the joint local correction stage, hand joint local features are constructed twice, so that the hand joint features contain inter-point relationships, enriching the hand joint feature information. The reconstructed features include structural information between joint points and positional reference information between joint points and their domain points, effectively improving the robustness of hand joint node estimation. We trained and tested our model on 3D point cloud datasets ICVL, NYU, and MSRA, and conducted complex scene experimental analysis on the InterHand2.6M dataset, while also developing virtual–real interaction applications. The experimental results show that the proposed method for hand joint estimation has improved accuracy compared to existing popular 3D point cloud gesture estimation algorithms, and has strong generalization ability. Next, we will further enhance the robustness and stability of the EdgeConv-based 3D point cloud gesture estimation method from the perspective of multimodal information fusion, and achieve widespread application of natural gesture interaction in more scenarios.

## Figures and Tables

**Table 1:** 3D point cloud gesture estimation dataset.

Dataset	Release Time	Number of Images	Category	Number of Joints	Labeling Method	Perspective	Image Size
ICVL	2014	17,604	10	16	semi-automatic	3	320 × 240
NYU	2014	81,009	2	36	semi-automatic	3	640 × 480
MSRA	2015	76,375	9	21	semi-automatic	3	640 × 480

**Table 2:** Comparison of total average errors between the method proposed in this article and other methods (unit: mm).

Algorithm	SHPR-Net	HandPointNet	Point-to-Point	HandFoldingNet	HandJoKe	Ours
ICVL	7.22	6.94	6.79	6.33	6.22	**6.29**
NYU	10.78	10.54	9.73	9.04	8.78	**8.42**
MSRA	7.76	8.51	7.86	7.71	7.52	**7.24**

**Table 3:** Comparison between InterHand2.6M dataset and existing algorithms.

Algorithm	APh/%	MPJPE/mm		MRRPE/mm
		Singe Hand	Two Hands	
PoseNet (2017) [33]	99.20	45.74	51.44	41.45
InterNet (2020) [34]	99.14	12.16	16.02	32.59
DIGIT (2021) [35]	99.15	11.32	15.57	30.51
Keypoint (2022) [36]	95.71	10.99	14.34	29.63
HDRNet (2022) [37]	96.84	8.51	13.12	27.46
GroupPoseNet (2022) [38]	99.02	9.10	12.82	31.37
Handformer2T (2024) [39]	97.62	**8.28**	10.72	**25.10**
mmWave-HGR (2025) [40]	61.53	32.87	46.32	68.94
BCPoseNet (2026) [3]	83.26	14.63	19.75	37.41
**Ours**	**99.05**	8.92	**10.32**	26.38

**Table 4:** Analysis of ablation experiment data (unit: mm).

Algorithm	MPJPE		MRRPE
	Singe Hand	Two Hands	
Global EdgeConv	16.85	21.32	47.32
Global EdgeConv + Initial Joint Estimation Module	13.16	17.07	34.17
Global EdgeConv + Initial Joint Estimation Module + Joint Correction Module	**10.43**	**12.67**	**29.14**

**Table 5:** Real-time comparison analysis between the method proposed in this article and other 3D point cloud methods.

Algorithm	SHPR-Net	HandPointNet	Point-to-Point	HandFoldingNet	HandJoKe	Ours
Time (ms)	21.4	14.4	16.7	11.9	28.5	19.1
Frame rate (FPS)	49.3	69.4	59.9	84.0	36.2	47.6

## Data Availability

The data are contained within this article.
